# Plasma kisspeptin levels are associated with insulin secretion in nondiabetic individuals

**DOI:** 10.1371/journal.pone.0179834

**Published:** 2017-06-21

**Authors:** Francesco Andreozzi, Gaia Chiara Mannino, Elettra Mancuso, Rosangela Spiga, Francesco Perticone, Giorgio Sesti

**Affiliations:** Department of Medical and Surgical Sciences, University Magna-Græcia of Catanzaro, Catanzaro (CZ), Italy; Consiglio Nazionale delle Ricerche, ITALY

## Abstract

To evaluate if plasma kisspeptin concentrations are associated with insulin secretion, as suggested by recent *in vitro* studies, independently of confounders. 261 nondiabetic subjects were stratified into tertiles according to kisspeptin values. Insulin secretion was assessed using indexes derived from oral glucose tolerance test (OGTT). After adjusting for age, gender, and BMI, subjects in the highest (tertile 3) kisspeptin group exhibited significantly lower values of insulinogenic index, corrected insulin response (CIR_30_), and Stumvoll indexes for first-phase and second-phase insulin release as compared with low (tertile 1) or intermediate (tertile 2) kisspeptin groups. Univariate correlations between kisspeptin concentration and metabolic variables showed that kisspeptin concentration was significantly and positively correlated with age, blood pressure, and 2-h post-load glucose, and inversely correlated with BMI, and waist circumference. There was an inverse relationship between kisspeptin levels and OGTT-derived indexes of glucose-stimulated insulin secretion. A multivariable regression analysis in a model including all the variables significantly correlated with kisspeptin concentration showed thar age (β = -0.338, *P<*0.0001), BMI (β = 0.272, *P<*0.0001), 2-h post-load glucose (β = -0.229, *P<*0.0001), and kisspeptin (β = -0.105, *P =* 0.03) remained associated with insulinogenic index. These factors explained 34.6% of the variance of the insulinogenic index. In conclusion, kisspeptin concentrations are associated with insulin secretion independently of important determinants of glucose homeostasis such as gender, age, adiposity, 2-h post-load glucose, and insulin sensitivity.

## Introduction

Kisspeptins are a family of peptides encoded by the *KISS1* gene [1–3]. *KISS1* was originally identified as a human metastasis suppressor gene (also named metastin) that had the ability to suppress melanoma and breast cancer metastasis [1]. The *KISS1* gene encodes for a hydrophobic 145-amino acid protein that is C-terminally amidated and proteolytically processed to form a 54-amino acid protein, named kisspeptin 54 (KP54) [1,2] as well as shorter peptides with the referred to with respect to their size–kisspeptin 10 (KP10), kisspeptin 13 (KP13) and kisspeptin 14 (KP14), respectively [4]. All these peptides share the same C-terminal 10 amino acid amidated sequence. Subsequent studies have shown that kisspeptins exert their action through binding to and activating a specific Gq/G11 protein-coupled receptor (GPCR) [3,5]. The GPCR GPR54 (also known as AXOR-12, and later renamed the kisspeptin 1 receptor, KISS1R) was identified to bind to and transmit the cellular action of secreted kisspeptins [3]. Kisspeptin-10, the common C-terminal decapeptide shared by all kisspeptin forms, is the minimum sequence necessary for GPR54 receptor activation [3,5]. In addition to the involvement in inhibiting cancer metastasis, kisspeptin-GPR54 signaling has an important role in the neuroendocrine control of the gonadotropin axis, and has an important role in the onset of puberty [6–11]. Kisspeptin has also been found to be associated with body mass index and indices of insulin resistance in women with polycystic ovary syndrome [12]. In addition, both kisspeptin and its receptor gene are expressed in placenta [1, 2, 5], and kisspeptin concentrations are elevated during pregnancy [12,13], suggesting that kisspeptin might play a role in restraining trophoblast invasion and regulating implantation and subsequent placental development [14–16].

Interestingly, a few studies have pointed to a potential role of the kisspeptin system in insulin secretion [17–21]. It has been shown that kisspeptin and GPR54 mRNAs are expressed in murine and human pancreatic islets, and in MIN6 pancreatic β-cell line [17]. However, the effects of kisspetins on insulin secretion have led to conflicting results. While some investigators reported that kisspeptin at nanomolar concentrations inhibits glucose-stimulated insulin secretion [18–20], others reported that micromolar kisspeptin concentrations stimulate glucose-stimulated insulin secretion [17, 20, 21]. Notably, in pancreatic islets isolated from mice lacking pancreatic *KISS1R* treatment with kisspeptin at nanomolar concentrations was unable to suppress glucose-stimulated insulin secretion as observed in control islets [20]. By contrast, kisspeptin at micromolar concentrations enhanced glucose-stimulated insulin secretion even in the absence of KISS1R, thus suggesting that kisspeptin at supraphysiologic concentrations stimulates insulin secretion independently of its specific receptor via unknown mechanism. Finally, using both animal and cellular models, it has been shown that glucagon stimulates hepatic expression of kisspeptin, which acts on pancreatic β-cell to suppress glucose-stimulated insulin secretion [20]. Whether these preclinical findings hold true in humans is unsettled. To the best of our knowledge, there is no information on the independent association between plasma kisspeptin concentrations and glucose-stimulated insulin secretion after adjustments for potential confounders. To address this issue, we examined the relationship between plasma kisspeptin concentrations and insulin secretion measured during an oral glucose tolerance test (OGTT) in a cohort of nondiabetic adult individuals.

## Materials and methods

### Study population

The study group consisted of 261 adult nondiabetic White individuals participating in the CAtanzaro MEtabolic RIsk factors (CATAMERI) study, an observational study assessing cardio-metabolic risk factors in individuals carrying at least one risk factor including overweight/obesity, hypertension, dyslipidemia, dysglycemia and family history for type 2 diabetes [22].

Exclusion criteria for the study population were: history of type 1 or type 2 diabetes mellitus, pregnancy status, end-stage renal disease, history of any malignant disease, gastrointestinal diseases associated with bleeding or malabsorption, chronic pancreatitis, history of alcohol or drug abuse, immunological diseases, acute infections or positivity for antibodies to hepatitis C virus (HCV) or hepatitis B surface antigen (HBsAg), and treatments able to modify glucose metabolism and inflammatory markers levels including corticosteroids, aspirin, glucose-lowering, lipid-lowering and antihypertensive therapy. All participants underwent anthropometrical evaluation and readings of clinic blood pressure obtained in the sitting position, after five minutes of quiet rest. After 12-h fasting, a 75g OGTT was performed with 0, 30, 60, 90 and 120 min sampling for plasma glucose and insulin. The study was approved by the local ethics committee (Comitato Etico Azienda Ospedaliera “Mater Domini”). Written informed consent was obtained from each subject in accordance with principles of the Declaration of Helsinki.

### Biochemical assays

Plasma kisspeptin concentration was measured with KISS 1 (112–121) Amide/Kisspeptin 10/Measin [45–54] Amide (Human) EIA KIT (EK-048-56, Phoenix Pharmaceuticals, Inc. Burlingame, CA, USA). Intra-assay variation was <10%; inter-assay variation was <15%, with minimum detectable concentration = 0.05 ng/ml.

Glucose, triglycerides, total and high density lipoprotein (HDL) cholesterol concentrations were determined by enzymatic methods (Roche, Basel, Switzerland). Plasma insulin concentration was measured with a chemiluminescence-based assay (Immulite^®^, Siemens Healthcare GmbH, Erlangen, Germany), and total serum IGF-1 was assayed by one-step sandwich chemiluminescence immunoassay (CLIA) after prior separation of IGF-1 from binding proteins on the Liaison® autoanalyzer (DiaSorin, Saluggia, Italy).

### Calculations

Insulin sensitivity estimated by the Matsuda index of insulin sensitivity (ISI) was calculated as follows: 10.000/square root of [FPG x fasting insulin] x [mean glucose x mean insulin during OGTT] [23]. Four indexes of glucose-stimulated insulin secretion were calculated from the OGTT data. Early phase of insulin secretion during an OGTT was estimated by the insulinogenic index as follows: Ins_30_-Ins_0_/Gluc_30_-Gluc_0_ (ΔIns30/ΔGluc30) where Ins_*y*_ and Gluc_*y*_ represent insulin and glucose values, respectively, at time *y* min during the OGTT. Stumvoll indexes for first and second phase insulin release were calculated using measurement of plasma glucose, and insulin every 30 min during an OGTT according to the formulas: first-phase = 1283 + 1.829 x Ins30–138.7 x Gluc30 + 3.772 x Ins0, and second-phase = 287 + 0.4164 x Ins30–26.07 x Gluc30 + 0.9226 x Ins0, respectively [24]. The corrected insulin response (CIR_30_) according to the formula: Ins_30_/(Gluc_30_ x (Gluc_30_−70)) [25]. To evaluate β-cell function the so-called disposition index was calculated as ΔIns30/ΔGluc30 x the Matsuda index.

### Statistical analysis

Variables with skewed distribution including triglycerides, and fasting insulin were natural log transformed for statistical analyses. Continuous data are expressed as means ± SD. Categorical variables were compared by χ2 test. ANOVA were used to compare differences of continuous variables between groups, as appropriate. Individuals were stratified into tertiles according to their plasma kisspeptin concentrations, and anthropometric and metabolic differences amongst groups were tested after adjusting for confounders using a general linear model. A multivariable linear regression analysis was performed in order to evaluate the independent contributions of plasma kisspeptin and other metabolic factors to insulin secretion. The variance inflection factor (VIF) was less than 2 in all the analyses indicating that multicollinearity among variables was not a problem in the multiple regression models. Two-sided *P* value <0.05 was considered statistically significant. All analyses were performed using the statistical package SPSS 22.0 for Windows (SPSS, IBM®, Chicago, IL).

A power calculation was performed at http://www.statisticalsolutions.net/pssZtest_calc.php and revealed a 90% power of detecting a 2.8 μU/ml per mg/dl difference in insulinogenic index or a 170 pmol/l difference in Stumvoll 1st phase index between two groups, with a two-sided test at a 5% significance level.

## Results

The mean age of the whole study sample was 47±13 years, 137 (52.5%) individuals were male, and mean BMI was 30.0±7.5 kg⁄m^2^. Biochemical and clinic features of the study sample stratified according to tertiles of kisspeptin value are shown in Table 1.

**Table 1:** Anthropometric and metabolic characteristics of the study subjects stratified according to tertiles of plasma kisspeptin values.

	Whole cohort	Tertile 1	Tertile 2	Tertile 3	*P*			
		(1)	(2)	(3)		1 vs 2	1 vs 3	2 vs 3
**Kisspeptin (*ng/ml*)**	**0.92±0.76**	**0.33±0.07**	**0.62±0.14**	**1.8±0.71**	**<0.0001**	**<0.0001**	**<0.0001**	**<0.0001**
Gender (Male/Female)	137/124	43/44	48/39	46/41	0.74	0.54	0.76	0.88
Age (*years*)	47.7±13.2	43.4±13.6	48.5±13.4	51.4±11.2	<0.0001	0.01	<0.0001	0.13
BMI (*kg/m2*)	30.1±7.6	30.7±8.9	30.8±8.1	28.4±4.8	0.15^a^	0.72 ^a^	0.14 ^a^	0.06^a^
Waist circumference (*cm*)	101.9±17	102±19	104±18	98±12	0.06 ^a^	0.36 ^a^	0.16 ^a^	0.02 ^a^
Systolic blood pressure (*mmHg*)	128±17	124±17	128±15	131±17	0.16	0.60	0.06	0.16
Diastolic blood pressure (*mmHg*)	79±10	76±10	79±10	82±10	0.006	0.20	0.002	0.04
Total cholesterol (*mg/dl*)	198±37	198±36	196±35	200±39	0.67	0.38	0.78	0.55
HDL (*mg/dl*)	51±14	51±13	51±15	51±13	0.68	0.70	0.63	0.38
Triglycerides (*mg/dl*)	122±72	114±55	122±74	127±82	0.62	0.91	0.44	0.36
Fasting Glucose (*mg/dl*)	97±14	95±13	97±13	99±14	0.70	0.87	0.43	0.51
2-h post-load glucose (*mg/dl*)	131±37	124±36	131±33	136±40	0.53	0.90	0.30	0.35
Glucose tolerance status (NGT/IGT)	159/102	55/32	54/33	50/37	0.71	0.88	0.53	0.63
Fasting Insulin (μ*U/ml*)	11±6	11±6	11±8	9±4	0.61	0.54	0.73	0.33
IGF-1(*ng/ml*)	164±59	170±63	164±59	158±54	0.80	0.80	0.68	0.51
Matsuda insulin sensitivity index (*mg x L*^*2*^ *x mmol*^*-1*^ *x mU*^*-1*^ *x min*^*-1*^)	81±46	80±46	76±47	86±44	0.88	0.73	0.87	0.62

Data are means ± SD. Insulin, triglycerides and hsCRP levels were log transformed for statistical analysis, but values in the table represent a back transformation to the original scale. Categorical variables were compared by χ^2^ test. Comparisons among the three groups were performed using a general linear model. *P* values refer to results after analyses with adjustment for age, gender, and BMI; ^a^*P* values refer to results after analyses with adjustment for age, and gender. BMI = body mass index; HDL = high density lipoprotein; NGT = normal glucose tolerance; IGT = impaired glucose tolerance;IGF-1 = insulin-like growth factor 1.

We observed no significant differences in geneder distribution across the three study groups. Subjects in the highest (tertile 3) kisspeptin group were older and tended to be leaner than individuals with low (tertile 1) or intermediate (tertile 2) kisspeptin groups. No significant differences among the three groups were observed for systolic blood pressure, total and HDL cholesterol, triglycerides, plasma IGF-1 levels, fasting and 2-h post-load glucose, fasting insulin, and insulin sensitivity as assessed by the Matsuda index (Table 1). The proportion of subjects with impaired glucose tolerance (IGT) did not differ between the three groups. Subjects in the highest (tertile 3) kisspeptin group exhibited significantly higher diastolic blood pressure as compared with low (tertile 1) or intermediate (tertile 2) kisspeptin groups when corrected for age, gender, and BMI,.

### Glucose-stimulated insulin secretion

Differences between the three study groups in glucose-stimulated insulin secretion assessed by OGTT-derived indexes are presented in Table 2.

**Table 2:** Insulin secretion indexes of the study subjects stratified according to tertiles of plasma kisspeptin values.

	Tertile 1	Tertile 2	Tertile 3	*P*			
	(1)	(2)	(3)		1 vs 2	1 vs 3	2 vs 3
Insulinogenic index (Δ*Ins30/*Δ*Gluc30*) (μ*U/ml per mg/dl*)	19.2±10.1	18.1±10.2	10.4±6.0	0.01	0.48	0.04	0.006
Stumvoll 1st phase index (*pmol/l*)	1281±724	1227±844	798±370	0.04	0.42	0.05	0.01
Stumvoll 2nd phase index (*pmol/l*)	338±191	327±193	230±81	0.03	0.36	0.06	0.01
CIR_30_	0.0066±0.007	0.0056±0.0004	0.0031±0.0019	0.03	0.88	0.03	0.02
Disposition index (Δ*Ins30/*Δ*Gluc30 x Matsuda index*)	1346±914	1177±917	831±575	0.05	0.92	0.04	0.03

Data are means ± SD. Comparisons among the three groups were performed using a general linear model. *P* values refer to results after analyses with adjustment for age, gender, and BMI. CIR_30_ = corrected insulin response

After adjusting for age, gender, and BMI, subjects in the highest (tertile 3) kisspeptin group exhibited significantly lower values of insulinogenic index, corrected insulin response (CIR_30_), and 1st and 2nd-phase insulin release estimated by Stumvoll indexes, as compared with low (tertile 1) or intermediate (tertile 2) kisspeptin groups (Table 2). Because the ability of β-cells to respond to an increment in glucose levels is affected by insulin sensitivity, we calculated the disposition index (ΔIns30/ΔGluc30 x the Matsuda index), and adjusted insulin secretion levels, assessed by the insulinogenic index, for the degree of insulin sensitivity, represented by the Matsuda index, in order to obtain a more precise measure of β-cell function. Subjects in the highest (tertile 3) kisspeptin group exhibited a significantly lower disposition index value as compared with low (tertile 1) or intermediate (tertile 2) kisspeptin groups (Table 2), when the data were adjusted for age, gender, and BMI,

Univariate correlations between kisspeptin concentration and anthropometric and metabolic variables in the whole study group are presented in Table 3.

**Table 3:** Univariate correlations between kisspeptin concentration and anthropometric and metabolic variables.

	Whole study group		Subjects with NGT (N = 159)		Subjects with IGT (N = 102)	
	Kisspeptin		Kisspeptin		Kisspeptin	
	*r*	*P*	*r*	*P*	*r*	*P*
Age (*years*)	0.18	0.007	0.17	0.01	0.07	0.22
BMI (*kg/m*^*2*^)	-0.12	0.04	-0.13	0.04	-0.11	0.11
Waist circumference (*cm*)	-0.12	0.04	-0.10	0.10	-0.13	0.08
Systolic blood pressure (*mmHg*)	0.09	0.09	0.07	0.16	0.05	0.30
Diastolic blood pressure (*mmHg*)	0.17	0.007	0.15	0.02	0.10	0.16
Total cholesterol (*mg/dl*)	0.08	0.10	0.03	0.36	0.12	0.11
HDL cholesterol (*mg/dl*)	-0.01	0.48	-0.06	0.19	-0.13	0.09
Triglycerides (*mg/dl*)	0.07	0.15	0.10	0.09	0.06	0.29
Fasting glucose (*mg/dl*)	0.07	0.14	0.05	0.27	0.01	0.49
2-h glucose (*mg/dl*)	0.12	0.03	0.12	0.06	0.01	0.49
Fasting insulin *(*μ*U/ml)*	-0.10	0.06	-0.16	0.02	-0.01	0.37
IGF-1(*ng/ml*)	-0.09	0.10	-0.10	0.11	-0.04	0.34
Matsuda Insulin Sensitivity index (*mg x L*^*2*^ *x mmol-*^*1*^ *x mU*^*-1*^ *x min*^*-1*^)	0.01	0.41	0.05	0.23	0.01	0.48
Insulinogenic index (Δ*Ins30/*Δ*Gluc30*)(μ*U/ml per mg/dl*)	-0.24	<0.0001	-0.25	0.001	-0.17	0.04
Stumvoll 1st phase index (*pmol/l*)	-0.21	0.001	-0.22	0.003	-0.16	0.05
Stumvoll 2nd phase index (*pmol/l*)	-0.20	0.002	-0.21	0.004	-0.15	0.05
CIR_30_	-0.23	<0.0001	-0.25	0.001	-0.16	0.05
Disposition index (Δ*Ins30/*Δ*Gluc30 x Matsuda index*)	-0.20	0.002	-0.22	0.003	-0.17	0.05

NGT = normal glucose tolerance; IGT = impaired glucose tolerance; BMI = body mass index; HDL = high density lipoprotein; IGF-1 = insulin-like growth factor 1; CIR_30_ = corrected insulin response.

Kisspeptin concentration was significantly and positively correlated with age, diastolic blood pressure, and 2-h post-load glucose, and inversely correlated with BMI, and waist circumference (Table 3). There was an inverse relationship between plasma kisspeptin levels and OGTT-derived indexes of glucose-stimulated insulin secretion including the insulinogenic index, the corrected insulin response (CIR_30_), the Stumvoll indexes for first-phase and second-phase insulin release, and the disposition index (Table 3). The inverse relationship between plasma kisspeptin levels and OGTT-derived indexes of glucose-stimulated insulin secretion was also maintained when subjects with normal glucose tolerance or impaired glucose tolerance (IGT) were analyzed separately (Table 3).

To evaluate the independent contribution of circulating kisspeptin levels to glucose-stimulated insulin secretion estimated by the insulinogenic index or by the disposition index, we built a model of a multivariable regression including all the above variables significantly correlated with kisspeptin concentration (Table 4).

**Table 4:** Multiple regression analysis with insulinogenic index of insulin secretion or disposition index as dependent variable.

	**Independent contributors**	**Standardized Coefficient β**	***P***
**Model** includes kisspeptin, gender, age, BMI, diastolic blood pressure, and 2-h post-load glucose.	Age	-0.338	<0.0001
	BMI	0.272	<0.0001
	2-h post-load glucose	-0.229	<0.0001
	Kisspeptin	-0.105	0.03
	**Independent contributors**	**Standardized Coefficient β**	***P***
**Model** includes kisspeptin, gender, age, BMI, diastolic blood pressure, and 2-h post-load glucose.	2-h post-load glucose	-0.365	<0.0001
	Age	-0.148	0.01
	Kisspeptin	-0.119	0.03

BMI = body mass index.

Comparison of standardized coefficients allowed the determination of the relative strength of each trait’s association with the insulinogenic index (listed from strongest to weakest): age (β = -0.338, *P<*0.0001), BMI (β = 0.272, *P<*0.0001), 2-h post-load glucose (β = -0.229, *P<*0.0001), and kisspeptin (β = -0.105, *P =* 0.03) (Table 4). These factors explained 34.6% of the variance of the insulinogenic index.

The variables independently associated with the disposition index were: 2-h post-load glucose (β = -0.365, *P<*0.0001), age (β = -0.148, *P =* 0.01), and kisspeptin (β = -0.119, *P =* 0.03) (Table 4). These factors explained 27.6% of the variance of the disposition index (Table 4).

## Discussion

Recently, a few preclinical research studies have hypothesized that kisspeptin may be involved in the regulation of insulin secretion [17–21]. However, these in vitro studies have led to mixed results with some studies showing an inhibitory effects of kisspeptin on glucose-stimulated insulin secretion [18–20] and others showing a stimulatory effects [17, 20, 21]. It is likely that these divergent findings are due the concentrations for kisspeptin used in the various experiments with kisspeptin concentrations in the nanomolar range acting as a suppressor of glucose-stimulated insulin secretion whereas micromolar kisspeptin concentrations acting as a stimulator of insulin secretion. To settle these controversies we decided to investigate the relationship between plasma kisspeptin levels and insulin secretion in a cross-sectional observational study including 261 nondiabetic volunteers Herein we provide evidences that kisspeptin is significantly associated with reduced glucose-stimulated insulin secretion. This association was not affected by the insertion of several potential confounding factors such as age, gender, adiposity, blood pressure, and 2-h post load glucose levels in the statistical model. When insulin secretion was adjusted for the prevailing degree of insulin sensitivity using the disposition index (insulin sensitivity x insulin secretion), β-cell function was significantly correlated with kisspeptin concentrations even after adjusting for age, sex, BMI, blood pressure, and 2-h post load glucose levels. To the best of our knowledge, this is the first study that unravels the relationship between plasma kisspeptin concentration and insulin secretion in humans. Importantly, this relationship was observed at kisspeptin concentrations (nanomolar) that has been show to inhibit insulin secretion in vitro [20].

A previous study has found an increased levels of kisspeptin in three type diabetic individuals as compared to three normal control [20] and, another study reported that plasma kisspeptin levels were negatively correlated with indices of insulin resistance in women with polycystic ovary syndrome [12]. In the present study, we did not observe any correlation between kisspeptin concentrations and index of insulin sensitivity. By contrast, we found an inverse relationship between BMI and kisspeptin concentrations in accord with prior findings in in women with polycystic ovary syndrome [12]. The mechanism by which kisspeptin affect body weight is unknown. Interestingly, studies with mice lacking the kisspeptin receptor (*Kiss1r* KO mice) have shown that adult *Kiss1r* KO females maintained on a standard chow diet displayed a marked increase in body weight as compared with wild type littermates [26]. Subsequent studies have shown that impaired kisspeptin signaling causes lower metabolism and energy expenditure, which thereby drive increased adiposity *Kiss1r* KO female mice [27]. Whether increased kisspeptin levels increase energy expenditure in humans is still undefined.

IGF-1 has been reported to induce the expression of *KiSS-1* gene in the hypothalamus [28] allowing the speculation that differences in plasma IGF-1 levels would be responsible for changes in kisspeptin levels. This finding that plasma IGF-1 did not differ amongst the tertile groups argue against this possibility.

Our study is fairly solid because of the relatively large sample size, the demographically homogeneous group of European ancestry, which equally comprised male and female individuals, all not affected by diabetes mellitus nor undergoing treatments able to modify glucose homeostasis. All study subjects have been subjected to an OGTT, thus we obtained both fasting and 2-hour post-load glucose values, which are required to assess glucose tolerance status. The biochemical determination of hormonal and metabolic variables has been performed in fresh blood samples rather than in stored samples,.

Notwithstanding, this study also suffers some limitations. Firstly, we did not have access to direct measures of β-cell function (using, for example, hyperglycemic clamp study or iv glucose tolerance tests). Instead, we used detailed, extensively validated proxy measures of insulin secretion which are derived using insulin and glucose levels from multiple time points during an oral glucose challenge, and thus encompass the contribution of of the incretin effect to insulin release. Furthermore, all the participants enrolled in our study were individuals carrying at least 1 risk factor for type 2 diabetes, who collectively represent a highly predisposed category of people for whom international guidelines recommend the adoption of preventive measures and testing for diabetes. In addition to this, our measurements of kisspeptin have been performed on fasting serum samples; which might not fully capture the plethora of kisspeptin effects on insulin secretion. Finally, because of the cross-sectional nature of the present study we do not have the power to draw conclusions about the causal relationship between kisspeptin concentrations and insulin secretion or to speculate the contribution of kisspeptin to the defects observed in the context of overt type 2 diabetes. Though the association between kisspeptin concentrations and glucose-stimulated insulin secretion was independent of age, adiposity, glucose tolerance, and insulin sensitivity, which are reportedly recognized as the main factors affecting insulin secretion, it is not possible for us to exclude the involvement of other mechanisms, which might simultaneously affect insulin secretion and be responsible for the elevation of circulating kisspeptin levels.

## Conclusion

Kisspeptin levels are inversely associated with insulin secretion. The results presented in this study are novel and we propose that the mechanisms linking kisspeptin and insulin secretion are independent from other major modulators of glucose homeostasis, including gender, age, adiposity, glucose tolerance and insulin sensitivity. For future research purposes, we look forward to longitudinal studies that will be able to reveal whether kisspeptin has the potential to contribute to the etiopathogenesis of type 2 diabetes, independently of confounding factors.
